# Effects of Alt-RAMEC protocol with facemask therapy in cleft lip palate patients in a sample of Pakistani population

**DOI:** 10.1186/s12903-023-03093-w

**Published:** 2023-06-17

**Authors:** Qurrat-ul-ain Sami, Batool Ali, Waqas Ahmed Farooqui

**Affiliations:** 1 Department of Orthodontics, Dr. Ishrat-Ul-Ebad Khan Institute Of Oral Health Sciences (DIKIOHS), Dow University of Health Sciences, Karachi, Pakistan; 2 Department of Orthodontics, Dow Dental Collage (DDC), Dow University of Health Sciences, Karachi, Pakistan; 3 Department of Research, School of Public Health, Dow University of Health Sciences, Karachi, Pakistan

**Keywords:** Alt-RAMEC expansion, Protraction headgear, Treatment of cleft lip palate, Maxillary Protraction, Early Class III treatment

## Abstract

**Objective:**

The objective of the study is to evaluate the skeletal, dentoalveolar and soft tissue changes before and after treatment with Alt-RAMEC protocol and protraction headgear in comparison to the controls.

**Material and methods:**

A quasi experimental study was conducted in the orthodontic department on 60 patients of cleft lip and palate. These patients were divided into two groups. Group I was the Alt-RAMEC group that underwent Alt-RAMEC protocol followed by facemask therapy while group II was the control group that underwent RME and facemask therapy. Total treatment time in both the groups was approximately 6 to 7 months. Mean and standard deviation was calculated for all the quantitative variables. Pre and post treatment changes between treatment and control groups were made using paired t-test. Intergroup comparison between treatment and control group was analyzed using independent t-test. Significance for all tests was predetermined at a *P*-value of  ≤ 0.05.

**Results:**

The Alt-RAMEC group showed significant forward movement of maxilla and improvement in the maxillary base. A remarkable improvement in SNA was seen. The overall outcome was better maxillo-mandibular relationship as shown by positive ANB values and angle of convexity. More effect on maxilla and least effect on mandible was notified with Alt-RAMEC protocol and facemask therapy. Improvement in transverse relationship was also evident in the Alt-RAMEC group.

**Conclusion:**

Alt-RAMEC protocol in combination with protraction headgear is a better alternative to treat cleft lip and palate patients in comparison to the conventional protocol.

## Background

Cleft lip and palate (CLP) is one of the most common craniofacial developmental anomalies involving the craniofacial region [1]. It occurs because the maxillary process, medial nasal process, and lateral nasal process fail to fuse in the initial 6 weeks of gestation. The incidence is almost 1 in 700 births in the USA and UK, consisting of 11% to 15% of all congenital dysplasia [2, 3]. Limited studies are done on cleft patients in Pakistan. According to a recent study, CLP is the most frequent birth defect in the Pakistani population. Clefts on the left side are more common than those on the right. Males are mostly affected by cleft lip (with or without palate), while females have predominantly isolated cleft palate. The most quoted risk factor in our part of the world is consanguineous marriage [2, 4].

A variety of treatment strategies have been proposed for CLP patients, but the main objective is better functional and esthetic outcome at the end of the growth and treatment period. Rapid maxillary expansion (RME) with facemask therapy has been a conventional treatment in subjects with CLP to correct the sagittal and transverse discrepancy by expanding the circum-maxillary sutures that facilitates in the protraction of maxilla [5]. Expansion not only corrects the posterior crossbite but also aligns the maxillary halves. It increases the alveolar cleft width and creates room for bone graft placement. Furthermore, it assists in the trans-operative procedure for suture closure of nasal mucosa.Therefore, it is necessary to correct the maxilla and protract it at the same time to improve the class III pattern [6]. The goal of rapid maxillary expansion is to separate the maxillary halves transversely and disarticulate the suture before filling of the alveolar cleft with bone graft [5].

Liou [7] designed a method of maxillary protraction for young individuals without the help of skeletal anchorage called the alternate rapid maxillary expansion contraction (Alt-RAMEC) protocol. According to him, the Alt-RAMEC protocol displaces the maxilla more anteriorly and disarticulates the circum-maxillary sutures more effectively than a single course of RME [8]. The maxilla, therefore, could be protracted more effectively. This was also agreed upon by Wang et al., [9] who concluded that the Alt-RAMEC procedure expanded the coronal (56.9% vs. 36.1%) as well as the sagittal circum-maxillary sutures (94.4% vs. 64.8%) quantitatively more than RME.

The purpose of the current study was to gather more evidence regarding the Alt-RAMEC protocol. Expansion along with a facemask is indicated in patients with CLP to correct the transverse and sagittal discrepancies of the maxilla. Our study aimed at using the Alt-RAMEC protocol in CLP patients and compare it with the conventional protocol to assess the outcome in a sample of the Pakistani population, as no data is available on this treatment regime in our population. This regime is considered as an advancement in the orthodontic field in comparison with the conventional protocol (RME and facemask therapy), and it can be an asset to both the orthodontist and the oral maxillofacial surgeon. Hence, the objective of this study was to evaluate the skeletal, dentoalveolar, and soft tissue changes before and after treatment with Alt-RAMEC protocol facemask therapy (Alt-RAMEC/FM) and rapid maxillary expansion facemask therapy (RME/FM) in CLP patients.

## Material and methods

**Fig. 1:**
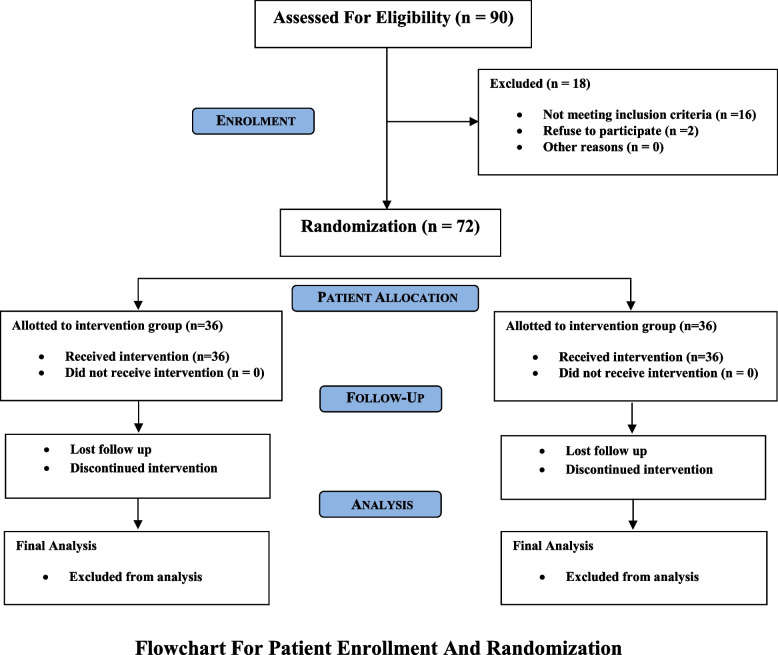
Flowchart for patient enrollment and randomization

A quasi-experimental study was conducted at the orthodontic department of Dow Dental College in January 2020 after ethical approval from the institution review board of Dow University of Health Sciences(Ref: IRB-883/DUHS/Approval/2017/148). Patients were recruited from multiple institutes of Karachi, including Civil Hospital, Patel Hospital, Saifi Hospital, Al-Mustafa Hospital, and Medilink Consultant Clinic, and called to the orthodontic department for treatment. The sample size was calculated using PASS version 15 (NCSS, Kaysville, Utah, USA) software with a 95% confidence interval, keeping the power of the study at 80% and the significance level at 0.05, using the values of the mean difference for the anteroposterior position of the maxilla with respect to the Sella-Nasion plane taken from the study conducted by Vieira et al. [10]. A total of 90 patients were approached via phone calls, and approximately 80 to 85 patients agreed to come for the treatment. Out of those patients, 72 were finally scrutinised to be a part of the study. Using a computer-generated random data sheet created with www.random.org, the CLP patients were randomly divided into two groups (ratio 1:1). Using consecutively numbered sealed envelopes, the allocation was hidden. Since the participants were oblivious of their placement in the treatment group, this study was single-blinded. Prior to the baseline, 72 participants were randomly assigned to either the control group (*n* = 36) and the Alt-RAMEC group (*n* = 36) by a second researcher. Out of which, 12 patients were excluded on the following basis: 2 patients presented with appliance breakage after a few visits; 5 patients had very poor oral hygiene and frequent de-cementation of the intra-oral expansion screw; 4 patients were lost to follow-up; and 1 patient was not compliant with facemask therapy. Thus, a sample size of 60 participants was included in the final analysis (30 in the Alt-RAMEC group, 30 in the control group) having equal number of unilateral and bilateral cleft lip and palate patients (Fig. 1). Each patient signed a written consent form before the start of the treatment.

The CLP patients were diagnosed and examined according to a strict inclusion criteria by a single operator. The inclusion criteria for this study was: patients having repaired lip and palate (unilateral and bilateral), 8–12 years old, having a negative overjet of greater than -2 mm, an ANB angle of less than 0, a SNMP angle of 32° ± 4°, CS-1 and CS-2 cervical maturation stage according to Baccetti [11], a transverse maxillary deficiency and a bilateral posterior crossbite of 3-5 mm. Patients with craniofacial syndrome, history of orthodontic treatment, incomplete clefts, isolated cleft lip and palate, mandibular prognathism, high angle (SNMP ˃ 36°), active periodontal disease, and missing, grossly carious, and mobile permanent molars, deciduous molars, and canines were excluded from the study.

Initially, pre-treatment records of all selected patients were collected. The records consisted of a lateral cephalogram and a dental cast. Lateral cephalogram was taken in the natural head position with teeth in centric occlusion and the Frankfort horizontal plane was kept parallel to the ground. Radiographs were taken with rigid head fixation and the same magnification factor using an ASAHI cephalostat (80 kVp, 10 mA, and 0.8 s exposure duration on 8 × 10 inch Kodak green film, Japan).

**Fig. 2:**
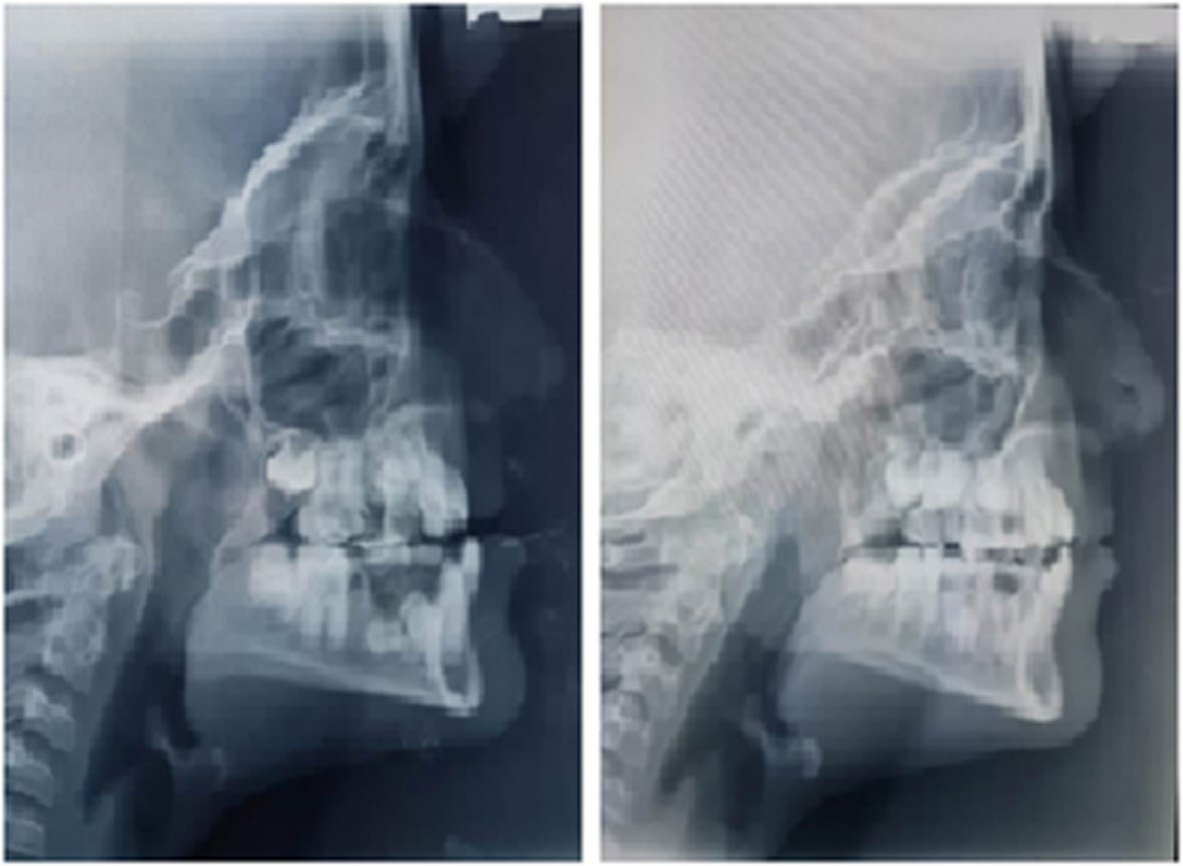
Sample representative treated case with Alt-RAMEC and facemask regime showing pre- treatment (left) and post-treatment (right) changes

An impression of the upper arch was made for the fabrication of an intraoral expansion appliance for each patient according to their respective dental cast. The appliance consisted of a bonded hyrax expansion screw [12] (palatal expansion screw = OrthoSource, USA; model = 820–009; 4 turns = 1 mm; size = 9 mm) placed parallel to the midline of the maxilla. Hooks were made on the buccal side of the appliance horizontally between the first and second deciduous molars with a vertical height of about 5 to 7 mm in the buccal sulcus. The anterior and posterior arms were bent to be embedded in the acrylic bite block. An acrylic bite block was fabricated that covered the buccal, occlusal, and palatal surfaces of the tooth to disocclude the dentition and facilitate transverse expansion. This appliance was cemented in the patient’s mouth with Glass Ionomer Cement-GIC (luting and lining type 1, GC Corp., Tokyo, Japan). Name, age, gender, and date of cementation of the appliance were documented for each patient. Parents were instructed to open the screw according to the treatment protocol. Group I was the Alt-RAMEC group in which parents were instructed to open the screw by 1 mm, i.e., 4 turns per day, during the first week and close it by 1 mm, i.e., 4 turns per day, the following week. This alternate opening and closing was repeated for seven consecutive weeks. After 7 weeks of alternate expansion and constriction, mild mobility of the maxilla was noted in all patients. Mobility was checked by supporting the forehead and bridge of the nose with one hand and holding the maxillary incisor with the other. The maxilla was then moved in an anterior and posterior direction to check for disarticulation [13]. Twenty six patients in Group 1 reported mild discomfort in the paranasal and zygomatic regions during the course of the Alt-RAMEC protocol. It was followed by facemask therapy for 4 to 5 months. In the control group, parents were instructed to open the hyrax screw by 1 mm per day, i.e., 4 turns per day, for 1 week, followed by face mask therapy for 5–6 months. The total treatment time in both groups was 6–7 months. A sample representative case is given in Fig. 2.

A petit type protraction facemask [12] (Model = F511-02, Type = Orthodontic adjustable headgear consisting of anterior bar, chin cup, and forehead padding, single bar; Zhejiang, China) was given to all the patients with elastics (Size = 5/16", Force = 14 oz-400 g, Ormco Co., USA) that were attached from hooks of the intraoral appliance to the left and right sides of the anterior bar with downward and forward pull of 30˚ to the occlusal plane. A force measuring gauge was used to measure the elastic force of approximately 500 g generated on each side. Patients were instructed to wear the protraction facemask for a minimum of 12 h per day. To ensure adequate wear time, patients maintained a written record of their daily facemask wear. Compliance was evaluated by hours of wear of the facemask and overjet reduction clinically. Facemask therapy continued until a positive overjet of 2 mm was achieved. At this stage, the treatment was stopped, and the expander and facemask were left in place for an additional 3 months for retention, following which post-treatment records were taken.

**Fig. 3:**
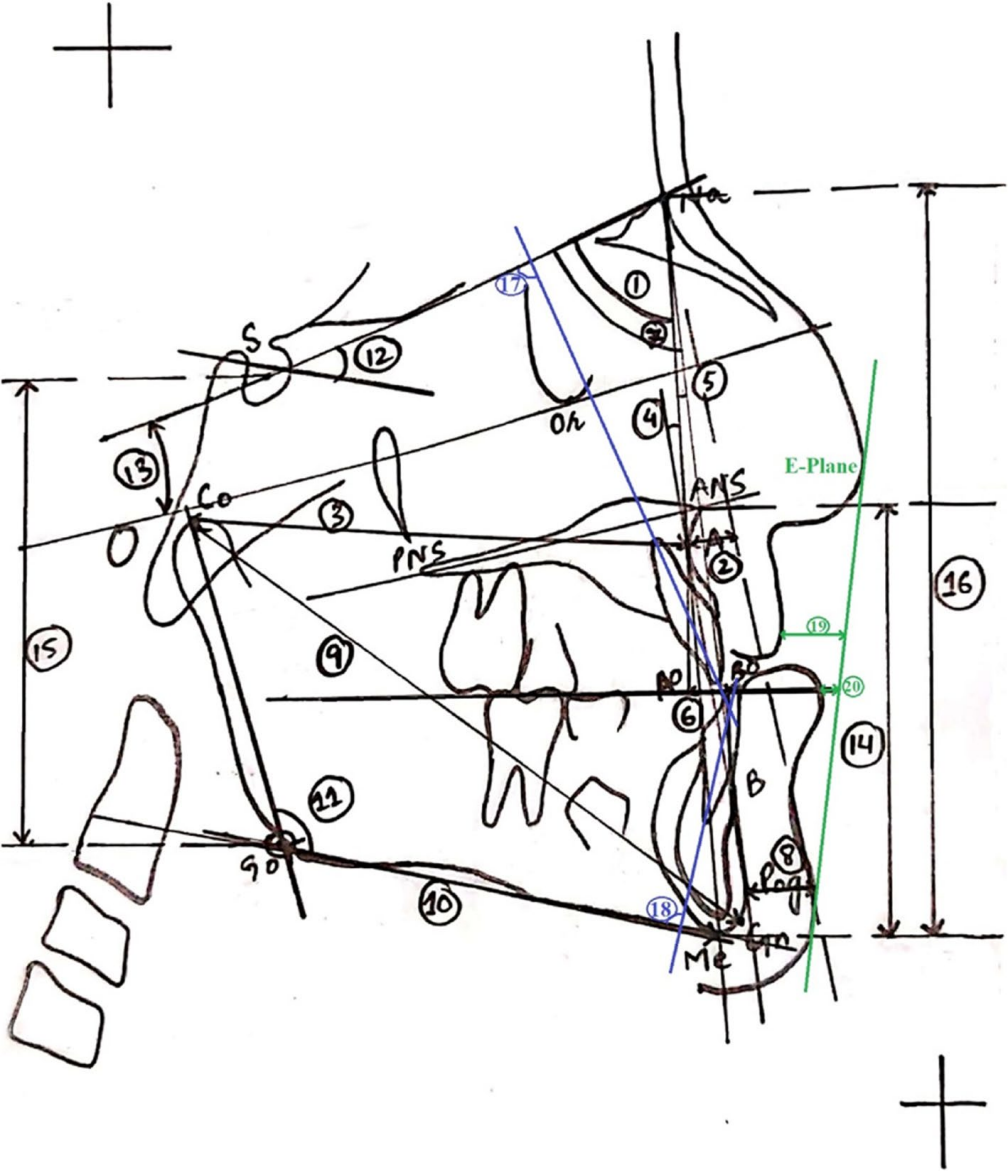
Skeletal, dental and soft tissue measurements* (1) SNA (2) Mc-A (3) Co-A (4) Angle of Convexity (5) ANB (6) AO-BO (7) SNB (8) Mc-Pog (9) Co-Gn (10) Go-Me (11) Gonial Angle (12) SNMP (13) SN-PP (14) LAFH (15) PFH (16) TFH (17) UISN (18) IMPA (19) UL E-PLANE (20) LL E-PLANE *SNA = Angle formed between Sella-Nasion plane (SN) and point A; Mc-A = Distance measured from McNamara line (Mc) to point A; Co-A = Distance measured from Condylion (Co) to point A; Angle of convexity = Angle formed at the intersection of nasion- point A to point A –Pogonion; ANB = Difference between SNA and SNB. It represents relative position of maxilla and mandible to each other; AO-BO = perpendicular lines were drawn from point A (AO) and point B (BO) to the occlusal plane and the distance was measured in millimeters; SNB = Angle formed between Sella-Nasion plane (SN) and point B; Mc-Pog = Distance measured from McNamara line (Mc) to Pogonion (Pog); Co-Gn = Distance measured from Condylion (Co) to Gnathion (Gn); Go-Me = Distance measured from Gonion point (Go) to Menton (Me); Gonial angle = Angle formed by tangents to the body of the mandible and posterior border of the ramus; SNMP = Angle formed between Sella-Nasion plane (SN) and mandibular plane (MP); SNPP = Angle formed between Sella-Nasion plane (SN) and palatal plane (PP); LAFH = vertical distance measured from anterior nasal spine (ANS) to Menton (Me); PFH = vertical distance measured from Sella turcica (S) to Gonion (Go); TFH = vertical distance measured from nasion point (Na) to Menton (Me); UI-SN = Angle formed between Sella-Nasion plane (SN) and upper incisor (UI); IMPA = Angle formed between mandibular plane and lower incisor; UL- E Plane = distance between the upper lip (UL) and esthetic plane (E- plane); LL- E Plane = distance between the lower lip (LL) and esthetic plane (E- plane)

**Fig. 4:**
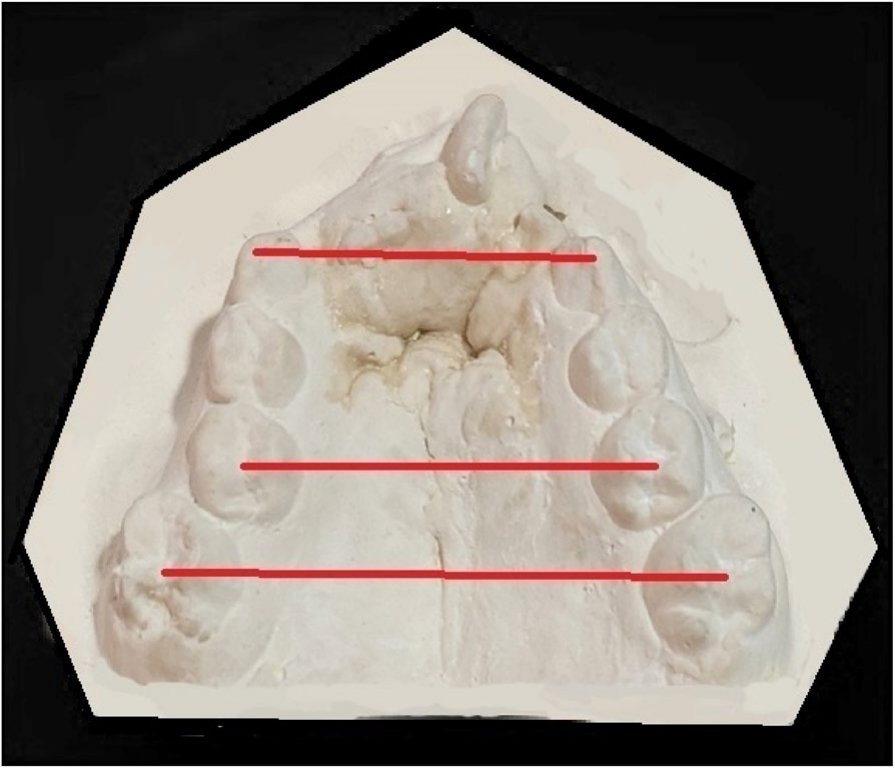
Transverse measurement * (1) ICW-D (2) IMW-D (3) IMW-P *ICW-D = Transverse distance measured from cusp tip of right canine to cusp tip of left canine; IMW-D = Transverse distance measured from central fossa of right deciduous second molar to central fossa of left deciduous second molar; IMW-P = Transverse distance measured from central fossa of right permanent first molar to central fossa of left permanent first molar

The pre- and post-treatment cephalograms of each patient were traced using acetate paper by the principal investigator. The skeletal, dentoalveolar, and soft tissue variables were measured on a lateral cephalogram and dental cast to evaluate changes before and after treatment. All the quantitative variables are elaborated in Figs. 3 and 4. A digital vernier calliper (0–150 mm ME00183, Dentaurum, Pforzheim, Germany) with an accuracy of 0.02 mm and reliability of 0.01 mm as per the manufacturer’s specification was used for calculating the dental cast measurements.

The collected data was then subjected to statistical analysis using IBM SPSS Version 22 software (IBM Inc., Armonk, NY, USA). The Shapiro–Wilk test was used to test the normality of the data, which showed a normal distribution; hence, parametric tests were applied. Means and standard deviations were calculated for all the quantitative variables. Pre- and post-treatment changes in the Alt-RAMEC group and control group were carried out using a paired t-test. An intergroup comparison between the Alt-RAMEC group and the control group was made using an independent t-test. The significance of all tests was predetermined at a *p-*value of 0.05.

## Results

Table 1 shows the skeletal, dental, and soft tissue changes achieved in the Alt-RAMEC group and control (RME/FM) group. The maxilla showed marked improvement in the sagittal direction in both the groups (*p*-value <0.001). Changes were more pronounced in the Alt-RAMEC group, where SNA was increased to 78.36˚ at the post-treatment stage, whereas the change was 77.3˚ in the control group. There was noteworthy anterior movement of the maxilla as well as forward movement of point A in the Alt-RAMEC group in contrast to the controls. The angle of convexity was also significantly better in the Alt-RAMEC group. The maxillo-mandibular relationship improved in both the groups, but it was more noticeable in the Alt-RAMEC group, as evident by the ANB angle and anteroposterior jaw relationship (AOBO) at the post-treatment stage. The vertical skeletal changes, i.e., SNMP, LAFH, and SNPP, were more distinctive in the control group, while they were not affected in the Alt-RAMEC group. There was a remarkable increase in the maxillary incisor inclination (UISN), while the lower incisor angulation was significantly reduced but still within the normal range in both the groups. On the other hand, the dentoalveolar measurements, i.e., inter-canine width of deciduous teeth (ICW-D), intermolar width of deciduous teeth (IMW-D), and intermolar width of permanent teeth (IMW-P), were markedly increased at the end of the treatment. Improvement in the control group was also evident, but the changes were more pronounced at the post-treatment stage in the Alt-RAMEC group. The soft tissue profile was also significantly improved in the Alt-RAMEC group; the upper lip moved forward by 2.40 mm,whereas the lower lip moved back by 1.55 mm.

**Table 1:** Skeletal, dental and soft tissue changes in Alt-RAMEC and control group

**Variable**	**Alt-RAMEC Group [(n) = 30]**			**Control Group [(n) = 30]**		
	**T0**	**T1**	***P*-value**	**T0**	**T1**	***P*-value**
	**Mean ± SD**	**Mean ± SD**		**Mean ± SD**	**Mean ± SD**	
**Skeletal Measurement**						
SNA (˚)	75.13** ± **3.15	78.36 ± 2.91	**<0.001**	75.13 ± 2.01	77.30 ± 2.08	**<0.001**
Mc-A (mm)	-5.20** ± **2.64	-1.98 ± 2.60	**<0.001**	-4.63 ± 2.23	-2.56 ± 2.28	**<0.001**
Co-A(mm)	68.83** ± **6.31	71.96 ± 6.32	**<0.001**	68.53 ± 4.89	70.60 ± 4.87	**<0.001**
Convexity Angle (˚)	-3.66** ± **3.91	4.26 ± 4.10	**<0.001**	-2.83 ± 3.30	2.63 ± 3.45	**<0.001**
ANB (˚)	-2.23** ± **0.72	1.80 ± 1.12	**<0.001**	-2.30 ± 0.83	1.16 ± 0.94	**<0.001**
AO-BO (mm)	-2.85** ± **2.54	0.85 ± 1.92	**<0.001**	-2.58 ± 1.88	-0.08 ± 1.88	**<0.001**
SNB (˚)	77.36** ± **3.16	76.56 ± 3.03	**<0.001**	77.10 ± 2.46	76.36 ± 2.35	**<0.001**
Mc-Pog(mm)	-7.73** ± **4.40	-7.90 ± 3.68	0.782	-7.76 ± 4.35	-7.96 ± 3.74	0.720
Co-Gn (mm)	91.76** ± **7.56	91.90 ± 7.14	0.816	90.80 ± 5.44	89.96 ± 5.29	**0.038**
Go-Me (mm)	57.03** ± **5.28	57.30 ± 4.89	0.433	56.93 ± 5.15	57.10 ± 4.86	0.509
Gonial angle (˚)	132** ± **5.27	131 ± 5.03	0.182	131.86 ± 5.11	132.3 ± 5.54	0.102
SNMP (˚)	34.93** ± **3.69	35.83 ± 4.00	**0.043**	34.26 ± 3.08	36.23 ± 3.63	**˂0.001**
SNPP (˚)	10.76** ± **2.93	10.20 ± 2.23	0.114	11.06 ± 3.00	10.36 ± 2.20	0.046
LAFH (mm)	55.73** ± **4.08	56.70 ± 3.94	**0.015**	54.63 ± 3.52	57.53 ± 3.76	**<0.001**
PFH (mm)	62.20** ± **10.37	61.30 ± 6.56	0.509	62.23 ± 10.35	61.53 ± 6.46	0.607
TFH (mm)	94.43** ± **12.14	98.03 ± 7.81	0.078	95.63 ± 9.76	96.8 ± 9.61	**0.006**
**Dentoalveolar Measurement**						
UISN (˚)	85.96 ± 9.60	93.40 ± 8.31	**<0.001**	86.60 ± 8.30	93.36 ± 7.40	**<0.001**
IMPA (˚)	86.36 ± 6.53	82.03 ± 6.54	**<0.001**	86.36 ± 6.53	82.03 ± 6.54	**<0.001**
ICW-D (mm)	24.76 ± 2.19	29.16 ± 1.89	**<0.001**	25.1 ± 1.70	28.16 ± 1.64	**<0.001**
IMW-D (mm)	34.30 ± 3.00	38.76 ± 2.29	**<0.001**	34.06 ± 2.77	37.3 ± 2.62	**<0.001**
IMW-P (mm)	41.90 ± 1.53	44.66 ± 1.58	**<0.001**	41.7 ± 1.62	43.5 ± 1.57	**<0.001**
**Soft tissue Measurement**						
UL-E plane(mm)	-5.08 ± 2.78	-2.65 ± 2.48	**<0.001**	-4.21 ± 2.00	-2.73 ± 2.18	**<0.001**
LL-E plane (mm)	1.20 ± 2.71	-0.35 ± 1.74	**<0.001**	0.56 ± 1.99	-0.26 ± 1.41	**<0.001**

*P*-value ≤ 0.05; Paired sample t-test; Total Sample size (N) = 60; Alt-RAMEC Group = Alt-RAMEC regime and facemask therapy; Control Group = RME and facemask therapy; T0 = Pre-treatment value = Measured at the start of treatment; T1 = Post-treatment value = Measured at the end of treatment

Table 2 represents an intergroup comparison of the mean difference in skeletal, dental, and soft tissue measurements between the Alt-RAMEC and the control group. The results indicated that the Alt-RAMEC protocol in combination with protraction headgear is better than conventional RME and facemask therapy in moving the maxilla forward (*p*-value ˂ 0.001). More sagittal improvement of the maxilla was noticed in the Alt-RAMECgroup in comparison to the controls. A remarkable movement of Point A and a better angle of convexity was evident in the Alt-RAMEC group. Contrary to the control group, the maxillo-mandibular relationship was significantly enhanced in the Alt-RAMEC group. Our result also showed greater enhancement in the dentoalveolar relationship in the Alt-RAMEC group, i.e., the change in inter-canine width of the deciduous teeth and the intermolar width of the permanent teeth. Therefore, it is evident from the results that the Alt-RAMEC protocol and facemask therapy, showed significantly better outcomes in skeletal, dental, and soft tissue measurements in contrast to the control group, i.e., RME and facemask.

**Table 2:** Mean difference in skeletal, dental and soft tissue measurements between Alt-RAMEC group and controls

**Variable**	**Mean Difference of Alt-RAMEC group** **(T1-T0)**	**Mean Difference of Control Group** **(T1-T0)**	
	**Mean ± SD**	**Mean ± SD**	***P*-value**^**¥**^
**Skeletal Measurement**			
SNA (˚)	3.23 ± 1.07	2.30 ± 0.98	**0.001**
Mc-A (mm)	3.21 ± 1.69	2.06 ± 0.73	**0.001**
Co-A(mm)	3.13 ± 2.30	2.06 ± 0.52	**0.016**
Angle of convexity (˚)	7.93** ± **3.19	5.46 ± 2.17	**0.001**
SNB (˚)	-0.80 ± 0.80	-0.73 ± 0.78	0.747
Mc-Pog(mm)	-0.16 ± 3.27	-0.2 ± 3.02	0.967
Co-Gn (mm)	0.13 ± 3.10	-0.83 ± 2.10	0.163
Go-Me (mm)	0.26 ± 1.83	0.16 ± 1.36	0.811
Gonial angle (˚)	-0.23** ± **0.93	0.43 ± 1.40	**0.034**
ANB (˚)	4.03 ± 1.03	3.46 ± 1.19	**0.054**
AO-BO (mm)	3.70 ± 2.36	2.5 ± 0.90	**0.012**
SNMP (˚)	0.90** ± **2.32	1.96 ± 1.92	0.137
SNPP (˚)	-0.56** ± **1.90	-0.70 ± 1.84	0.783
LAFH (mm)	0.96 ± 2.04	2.9 ± 1.72	**0.000**
PFH (mm)	-0.90 ± 7.36	-0.7 ± 7.36	0.916
TFH (mm)	3.60 ± 10.77	1.16 ± 2.16	0.460
**Dentoalveolar Measurement**			
UISN (˚)	7.43 ± 7.46	7.10 ± 6.79	0.857
IMPA (˚)	-4.33 ± 5.27	-4.33 ± 5.27	1.000
ICW-D (mm)	4.40 ± 0.96	3.06 ± 0.58	**<0.001**
IMW-D (mm)	4.46 ± 2.09	3.23 ± 1.07	0.006
IMW-P (mm)	2.27 ± 0.67	1.80 ± 0.66	**<0.001**
**Soft tissue Measurement**			
UL-E plane (mm)	2.40 ± 1.25	1.48 ± 0.62	**<0.001**
LL-E plane (mm)	-1.55 ± 1.90	-0.83 ± 1.07	0.078

*P*-value ≤ 0.05; ^**¥**^Independent sample t-test; Sample size (n) for each group = 30; Alt-RAMEC Group = Alt-RAMEC regime and facemask therapy; Control Group = RME and facemask therapy; T0 = Pre-treatment value = measured at the start of treatment; T1 = Post-treatment value = measured at the end of treatment

## Discussion

This study was conducted to assess the effectiveness of the Alt-RAMEC treatment in comparison to the control group followed by maxillary protraction in individuals with cleft lip and palate who were developing a class III skeletal malocclusion. Immediate results of Alt-RAMEC treatment revealed a noticeable improvement in the position of the maxilla, with a backward movement of the mandible, and a better maxillo-mandibular relationship. Researchers reported that the implementation of the Alt-RAMEC protocol with facemask therapy in class III malocclusion has proven to be more efficient in treating maxillary prolapse than traditional facemasks combined with regular RME [14]. Canturk et al. [15] examined the effectiveness of the facemask used before and after the 8-week Alt-RAMEC regimen. Prior to or in conjunction with the facemask, the Alt-RAMEC protocol was not statistically different, although the maxilla was significantly more prominent in both groups.

Our study showed skeletal as well as dental changes at the post-treatment stage in both groups. According to the research conducted in Italy, the differences in measurements of SNA, SNB, and ANB with the Alt-RAMEC protocol were 3.43˚, -0.42˚, and 3.77˚, which were comparable to the measurements found in our study of 3.23˚, -0.80˚, and 4.03˚, respectively [16]. Another study conducted at a university in Marmara also supported our result for SNA, SNB, and ANB in the Alt-RAMEC group, as they had similar records of 2.71˚, 0.35˚, and 2.36˚, respectively [17]. The Alt-RAMEC protocol and FM was found to induce an increase in SNA by 3.43° ± 1.44°, an increase in ANB by 3.77° ± 1.33°, and a decrease in SNB angle of -0.42° ± 1.97° in UCLP patients [18]. CLP patients had a deficient maxilla with a concave facial profile. Improvement in SNA, forward movement of the maxilla, and slight backward movement of the mandible contributed to an improvement in the sagittal relationship as well as the angle of convexity [19]. Our study reported a significant change in the difference in angle of convexity of 7.93˚ at the end of the treatment in the Alt-RAMEC group. Our result was better than the findings reported by Halicioglu [20], Liu [21] and Singh et al. [1]. These findings pertaining to the angle of convexity explained that a better soft tissue profile was achieved in comparison to the conventional protocol. A clinician in Turkey investigated the outcome of FM therapy supported with miniplates after the Alt-RAMEC regime in class III patients. All patients were subjected to an 8-week expansion constriction (Alt-RAMEC) protocol followed by facemask therapy. The cephalometric results showed that the maxilla moved forward by 2 mm with a 0.8˚ anticlockwise rotation and no proclination of the incisors. In the mandible a clockwise rotation of 1.2˚ with a decrease in the inclination of the mandibular incisors by 2˚ was observed [22]. Yatabe et al. [23] also documented soft tissue alterations associated with bone anchored maxillary protraction. The midface, upper lip, and cheeks had substantial sagittal displacement. Negative sagittal alterations in the lower lip and chin revealed that the soft tissue growth was constrained with backward displacement in this region.

The purpose of the Alt-RAMEC protocol was to decrease the dentoalveolar effects and achieve more skeletal effects in a short period of time as it allowed osseous mobility through alternate opening and closing of the RME appliance for 7–9 weeks without unwanted expansion [16]. The alternate rapid maxillary expansions and constrictions (Alt-RAMEC protocol) accompanied by a facemask have been able to succeed in disarticulating the circum-maxillary suture for better protraction of the maxilla. The Alt-RAMEC method opened the maxillary centre of rotation at the location of the posterior nasal spine, allowing the tuber maxillae to extend forward without maxillary resorption. As a consequence, the sutures were significantly better mobilised without any resistance [8]. A literature survey suggested that the Alt-RAMEC regimen helped circum-maxillary suture disarticulation, with an increase in the maxillary protraction, in a much shorter time [6, 16, 19, 24, 25]. When the maxillary sutures were activated using the Alt-RAMEC approach, the maxilla advanced approximately twice as much as with typical RME or FM therapy. When assessed, the maxilla extended forward, anteriorly, significantly further, which relieved the patients from needing a future orthodontic and orthognathic surgery by overcompensating the maxilla's position [26, 27]. A CBCT study evaluated the effects of the Alt-RAMEC procedure on the maxilla, soft tissue, and airway indicated a slight forward (0.89 mm) and downward (0.92 mm) movement of point A [28]. They also reported that expansion improved other structures of the face, including the nasal bone, zygomaticomaxillary suture, and zygomaticotemporal suture (*p*-value ≤ 0.05). The width of the nasal bone was also increased. The soft tissues of the paranasal area showed significant changes, and airway volume increased [28]. Another CBCT study on CLP patients quoted that maxillary protraction combined with an Alt- RAMEC expansion produced better outcomes than maxillary protraction alone. The anterior landmarks of the maxilla, such as point A and ANS, were shifted further forwardly and the maxilla was also translated in a clockwise direction [1]. The length of the maxilla (Co-A) seemed to increase with the Alt-RAMEC protocol in all aforementioned research studies by approximately 4.13 mm [29]. According to a randomised clinical trial with the aim of protracting the maxilla and comparing RME versus Alt-RAMEC protocols, the maxillary length increased by 3.04 mm, the maxilla moved forward by SNA 2.67° and Mc-A showed an improvement of 2.48 mm in the Alt-RAMEC group [21]. This can be compared to our study, where Co-A changed by 3.13 mm with the sagittal movement of the maxilla by 3.21 mm (Mc-A) in the Alt-RAMEC group. The maxilla was significantly protracted by 1.87° ± 1.06°, with an improvement in the jaw relationship of 3.95 ± 0.57 and Wits of 5.15 ± 1.51 mm [13]. Another study reported an improvement in Wits appraisal (AO-BO) of 2.0 mm; all these results were in concordance with our result of 3.70 mm ± 2.36 [30]. The soft tissue changes regarding the Alt-RAMEC protocol have been studied in previous studies [13, 31]. Both the upper lip and lower lip showed a better soft tissue profile; the upper lip moved forward by 2.40 mm while the lower lip moved back by 1.55 mm in the Alt-RAMEC group, which is in accordance with the aforementioned studies.

The Alt-RAMEC protocol was helpful in improving the transverse measurement of the inter-canine region, inter-molar region of deciduous teeth, and intermolar region of permanent teeth. A recent study on BCLP patients comparing maxillary expansion with differential opening (EDO) with conventional RME showed intercanine width increased more with a difference of 3.63 mm with minimal buccal tipping with EDO when compared with conventional hyrax expanders [24]. In a study conducted in Turkey, increased inter-canine, inter-premolar, and intermolar widths were seen using the Alt-RAMEC protocol with FM [25]. Our study also reported an increase in the inter-canine, inter-premolar, and inter-molar widths by 4.40 mm, 4.46 mm, and 2.27 mm, respectively, in the Alt-RAMEC group, which is more than the studies mentioned above. This showed a good transverse relationship after the treatment with the Alt-RAMEC protocol. Recently, a randomised clinical trial conducted on BCLP patients via CBCT confirmed a lesser buccal inclination of canines with the Alt-RAMEC protocol with a 3.63 mm increase in inter-canine width [24]. Additionally, a systematic review explained that this protocol produced a remarkable increase in the transverse measurements of the mid-palatal sutures, and more anteriorly as compared to posteriorly [8, 24].

Further longitudinal research is needed to evaluate the outcome of reverse pull headgear on individuals with CLP in greater depth, i.e., through a three-dimensional evaluation rather than a two-dimensional one. Additionally, long-term follow-up of these patients needs to be done to better understand the stability of the treatment protocol.

### Strengths and limitations of the study

To the best of our knowledge, no similar research has ever been conducted in Pakistan, specifically directed towards CLP patients. In previous research conducted globally, either UCLP or BCLP patients had been included. However, this study accommodated an equal number of UCLP and BCLP patients, as the literature review showedthat the treatment protocols of both are the same. Both types of patients showed advancement of the maxilla, improvement in the maxillary length, posterior mandibular movement, and a better maxillomandibular relationship. This research helped boost the patient’s confidence as well as self-esteem through improvements in facial aesthetics and soft tissue profile as the study progressed.

The biggest limitation to our study was accumulating CLP patients at a single centre from multiple centres across Karachi. However, this was overcome with the help of a plastic surgeon based in the city. Another limitation was patient compliance since this study gathered data from young children. To overcome this limitation, the researcher kept them motivated by explaining the importance of this treatment. Additionally, seeing the great aesthetic enhancement and improvement in facial profile helped motivate the patients even more.

## Conclusion

The Alt-RAMEC protocol and facemask therapy in CLP patients have been shown to have considerable positive outcomes in cleft lip and palate patients with respect to changes in the skeletal, dental, and soft tissue measurements in comparison to the conventional protocol. Most of the changes observed in this study were in the maxilla. A significant protraction of the maxilla was accomplished. There was a remarkable forward movement of the point A with a better maxillo-mandibular relationship. Anterior movement of the maxilla was evident with improvement in angle of convexity in the Alt-RAMEC group. The mandible showed a slight decrease in SNB angle. With respect to dentoalveolar measurement, changes were more pronounced in the Alt-RAMEC group. The increase in width of the inter-canine and intermolar regions of the deciduous dentition and the intermolar region of the permanent dentition was more evident in the Alt-RAMEC group.

## Data Availability

The datasets used and analysed during the current study are available from the principal investigator (corresponding author) on reasonable request.

## Abbreviations

Alt-RAMEC: Alternate rapid maxillary expansion and constriction
ANB: Difference between SNA and SNB
AO-BO: Witt’s appraisal (perpendicular lines drawn from point A (AO) and point B (BO) to the occlusal plane and the distance between the lines was measured)
BCLP: Bilateral cleft lip and palate
CLP: Cleft lip and palate
CBCT: Cone beam computed tomography
Co-A: Anterio-posterior length of the maxilla
Co-Gn: Mandibular base length
EDO: Maxillary expansion with differential opening
FM: Facemask
GIC: Glass Inomer Cement
Go-Me: Mandibular body length
ICW-D: Intercanine width
IMPA: Angle formed between mandibular plane and lower incisors
IMW –D: Intermolar width of deciduous teeth
IMW –P: Intermolarwidth of permanent teeth
LAFH: Lower anterior facial height
LL- E-Plane: Distance measured from lower lip to Esthetic plane
Mc-A: Distance measured from McNamara line to point A
Mc-Pog: Distance measured from McNamara line to Pogonion
OPG: Orthopantomogram
PFH: Posterior facial height
RME: Rapid maxillary or palatal expansion
SNA: Angle formed between Sella-Nasion plane and point A
SNB: Angle formed between Sella-Nasion plane and point B
SNMP: Angle formed between Sella-Nasion plane and mandibular plane
SN-PP: Angle formed between Sella-Nasion plane and palatal plane
TFH: Total facial height
UCLP: Unilateral cleft lip and palate
UI-SN: Angle formed between Sella-Nasion plane and upper incisors
